# A Sight-Threatening Genetic Disorder: A Rare Ocular Manifestation of Von Hippel-Lindau Syndrome

**DOI:** 10.7759/cureus.97698

**Published:** 2025-11-24

**Authors:** Sue-Zian Goh, Wan-Hazabbah Wan Hitam, Hanisah Abdul Hamid, Rosnita Alias

**Affiliations:** 1 Department of Ophthalmology and Visual Science, School of Medical Sciences, Universiti Sains Malaysia, Kubang Kerian, MYS; 2 Ophthalmology Clinic, Hospital Sultan Abdul Halim, Sungai Petani, MYS

**Keywords:** exudative retinal detachment, haemangioblastoma, retinal haemangioblastoma, vhl, von hippel-lindau

## Abstract

Von Hippel-Lindau (VHL) syndrome is a rare inherited genetic disorder caused by mutations in the VHL tumor suppressor gene on chromosome 3. It leads to the development of multiple benign and malignant tumors in various organs.

This report details a rare case of ocular VHL syndrome in an 11-year-old girl. She presented with progressive vision loss in her right eye and a family history of blindness and brain tumors. On examination, her right eye showed almost total retinal detachment and severe vision impairment, while her left eye had multiple retinal hemangioblastomas with subretinal fluid but preserved visual acuity.

The patient was diagnosed with VHL syndrome and treated with laser photocoagulation for the retinal hemangioblastomas. After several treatments, her vision in the left eye remained stable at the six-month follow-up.

While VHL syndrome typically presents in late teens to early adulthood, this case highlights the importance of early diagnosis and continuous screening, especially given the condition's genetic nature and potential for multiorgan involvement.

## Introduction

Von Hippel-Lindau (VHL) syndrome is a rare condition that is inherited in an autosomal dominant pattern and associated with germline mutations in the VHL tumour suppressor gene, leading to the development of multiple tumours. The mean age at onset of VHL disease is 26.3 years. Retinal angioma was the first manifestation (43%), followed by cerebellar haemangioblastoma (39%) and renal cell carcinoma (10%). VHL is frequently associated with cerebellar haemangioblastoma (59%), retinal angioma (59%), renal cell carcinoma (28%), spinal haemangioblastoma (13%), and phaeochromocytoma (7%). The mean age of diagnosis for renal cell carcinoma was 44.0 years, while cerebellar haemangioblastoma was at 29.0 years and retinal angioma at 25.4 years. Mean age at death was 41 years, with renal cell carcinoma being the leading cause of death, followed by cerebral haemangioblastoma [1]. This report details a rare case of VHL in an 11-year-old girl who presented with retinal hemangioblastomas. This case underscores the critical importance of early screening and intervention to prevent severe complications, including blindness.

This article was previously presented as a poster at the 7th Asia Pacific Tele-Ophthalmology Society Symposium (APTOS) on September 3, 2022.

## Case presentation

An 11-year-old girl with no significant medical history presented with painless, progressively worsening blurring of vision in her right eye over the past month. She reported no additional symptoms such as diplopia, redness, photophobia, floaters, or eye pain, and had no signs of increased intracranial pressure. Her family history was significant for early-onset blindness and brain tumors: her mother lost her vision at age 16 and passed away at 34 due to a cerebellar cystic lesion and obstructive hydrocephalus, while her maternal aunts and grandmother also experienced early blindness and died from brain tumors.

On examination, the visual acuity in the right eye was light perception, while the left eye had 6/9 vision. The right eye exhibited a positive relative afferent pupillary defect and 15 degrees of exotropia. Additional optic nerve functions, including color vision, light brightness, and red saturation, were also impaired in the right eye. There was no proptosis, pain on eye movement, or limitation of extraocular muscle movements. Both anterior segments and intraocular pressure were normal.

**Figure 1:**
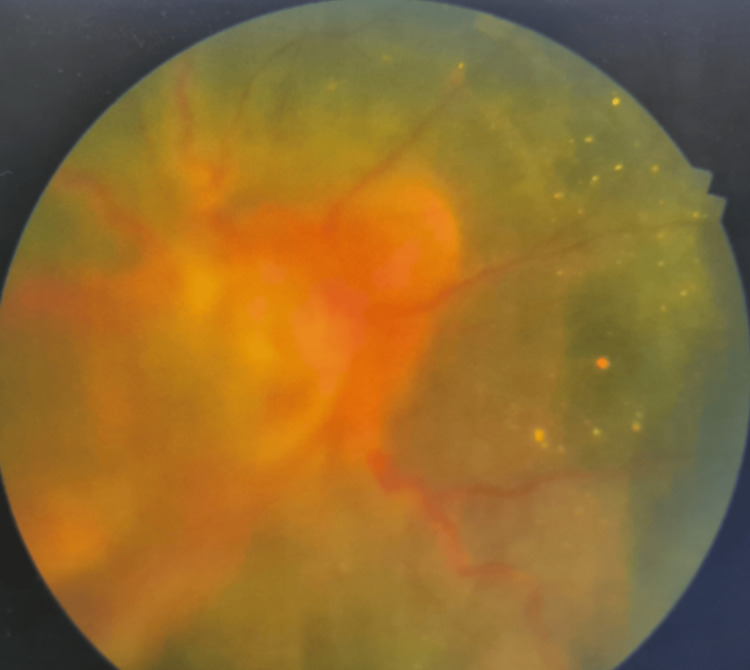
Fundus of the right eye with almost total exudative retinal detachment, sparing only the supero-nasal quadrant of the retina

Fundoscopy revealed almost total retinal detachment in the right eye, with only the supero-nasal quadrant remaining attached (Figure 1).

**Figure 2:**
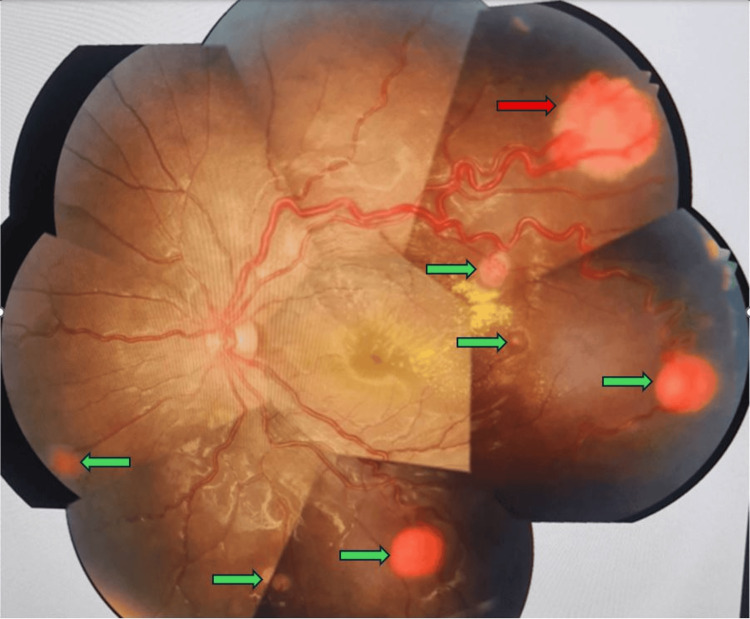
The left eye fundus displaying multiple retinal haemangioblastomas (green arrows) in the peripheral and midperipheral retina with subretinal exudate and dilated, tortuous veins in addition to one large retinal haemangioblastoma (red arrow)

**Figure 3:**
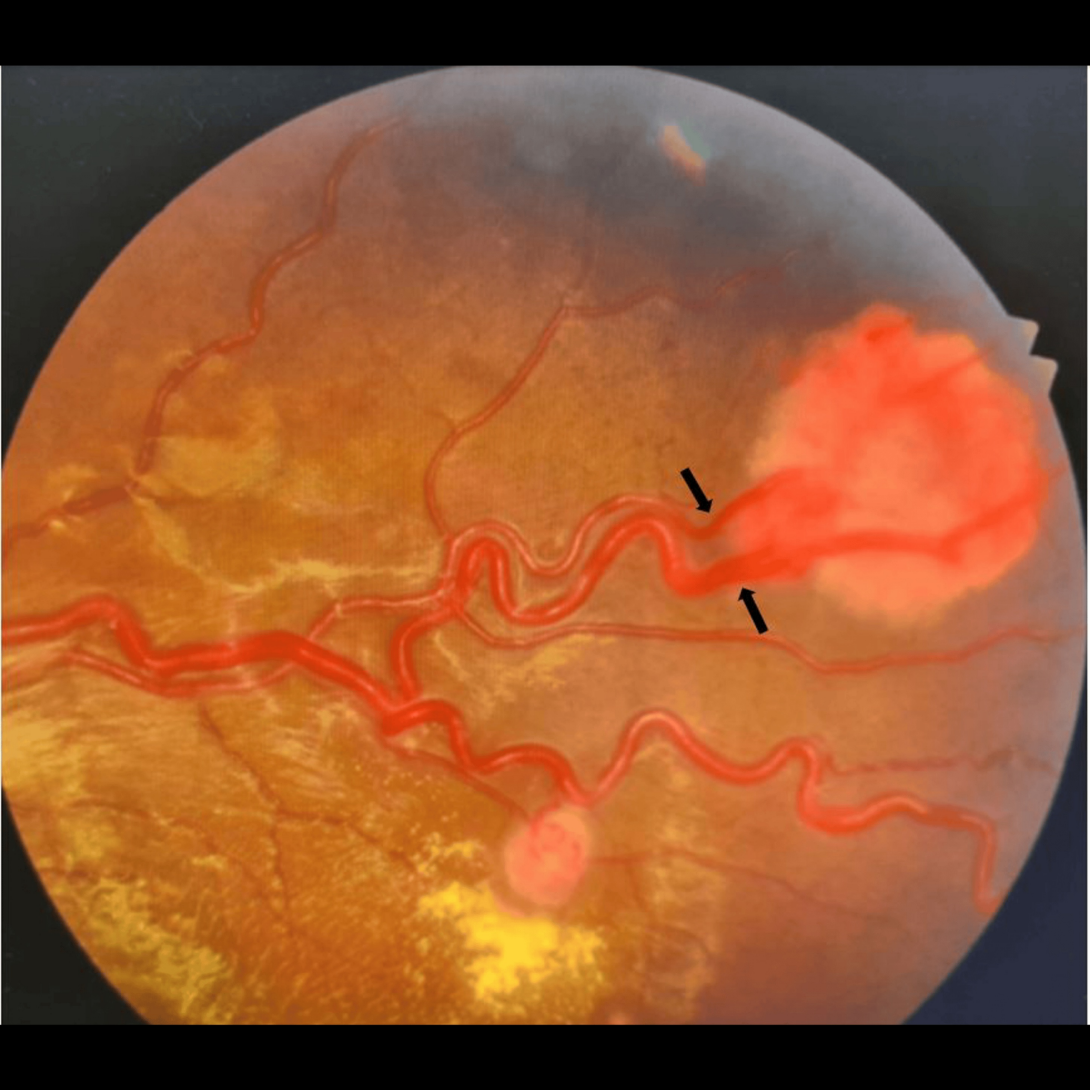
Large retinal haemangioblastoma located at the supero-temporal quadrant of the retina with two feeder vessels (black arrow)

In the left eye, multiple retinal hemangioblastomas were observed in the peripheral and mid-peripheral retina, accompanied by subretinal fluid and exudate. The optic disc and macula appeared normal, but there were dilated and tortuous veins in all quadrants (Figures 2-3).

**Figure 4:**
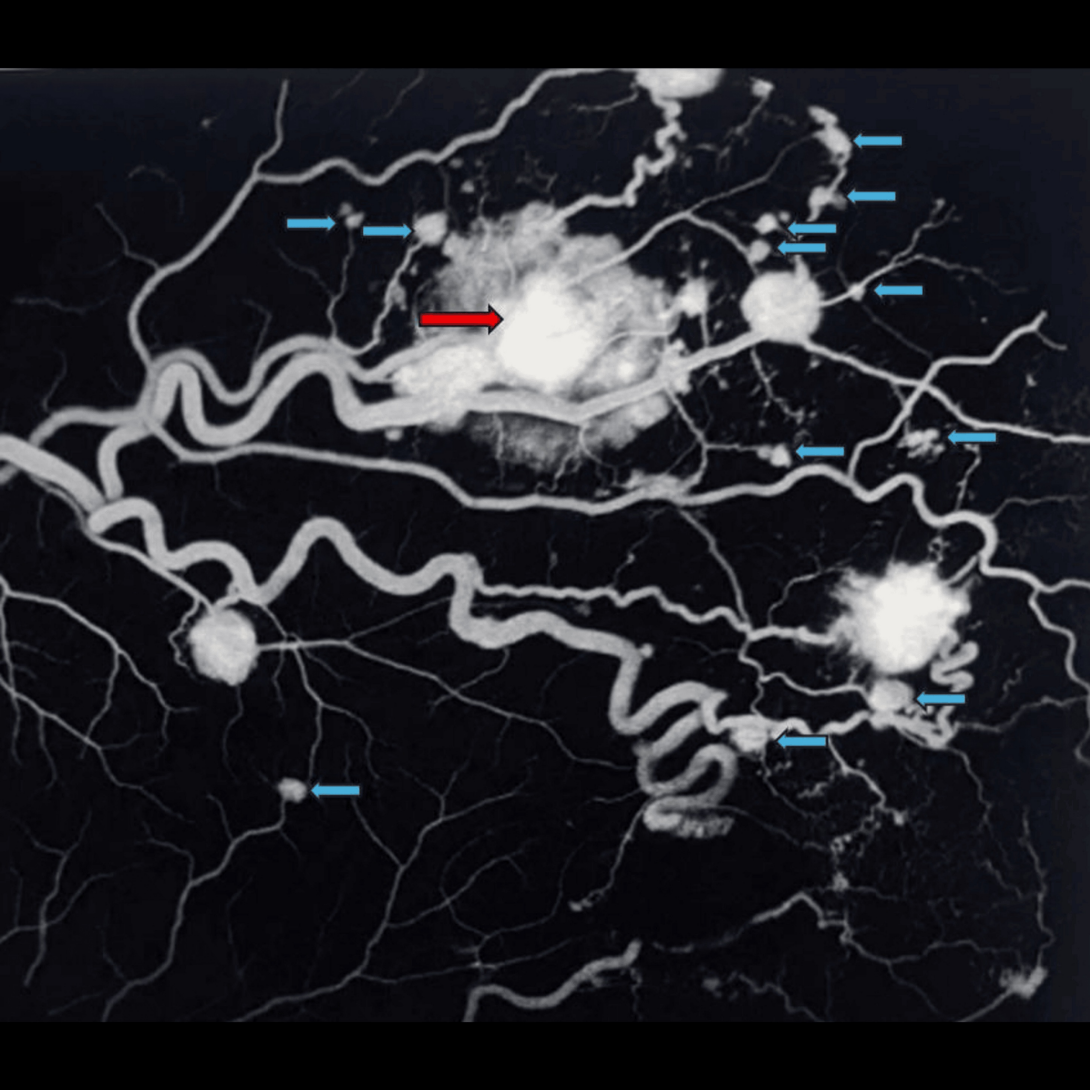
Fundus fluorescein angiography of the supero-temporal quadrant of retina showing one large retinal haemangioblastoma (red arrow) and multiple small subclinical retinal haemangioblastomas that were not visible on funduscopy (blue arrows)

Fundus fluorescein angiography showed additional subclinical retinal hemangioblastomas and a few leaking hemangioblastomas (Figure 4).

Systemic examination was unremarkable, and vital signs were stable. No abdominal mass or lymphadenopathy was noted. Neurological examination was normal. Brain CT was normal, and spinal MRI showed no lesions. Abdominal ultrasound did not reveal any intrabdominal or renal tumors. Given the positive family history of brain tumors and retinal hemangioblastomas, the diagnosis of VHL syndrome was established. Laser photocoagulation of the retinal hemangioblastomas was initiated, and the patient’s vision remained stable at the six-month follow-up with close monitoring.

## Discussion

VHL syndrome is a rare inherited disorder caused by mutations in the VHL gene located on chromosome 3p. It follows an autosomal dominant pattern of inheritance, characterized by a complex genetic locus and allelic heterogeneity. The prevalence of VHL syndrome ranges from 1 in 38,951 to 1 in 53,000 individuals [1,2].

While VHL syndrome commonly presents in late teens to early adulthood, our patient presented in her early teens. This emphasizes the need for early diagnosis and continuous screening, especially given the genetic nature of the condition and its potential to impact multiple organs. The syndrome's most common manifestations include retinal and central nervous system hemangioblastomas [1]. Approximately 84% of all retinal hemangioblastomas and about 30% of cerebellar hemangioblastomas are associated with VHL syndrome [3].

Individuals with VHL syndrome face significant risks, including a high probability of developing renal cell carcinomas, estimated at up to 40%. Additionally, they are prone to developing visceral cysts, such as those affecting the kidneys, pancreas, and epididymis [1]. The risk of developing retinal hemangioma in VHL patients is less than 1% before the age of five and increases to 5% before the age of 10 [1]. Visual loss due to retinal angiomas affects approximately 35% of all gene carriers and rises to 55% by age 50 in patients with retinal angiomas [4].

Untreated retinal angiomas can lead to complications such as retinal detachment, exudation of fluid, and subsequent visual loss [1]. Small retinal hemangioblastomas are often effectively treated with photocoagulation, while larger ones may require cryotherapy for successful management [1]. The tumors associated with VHL syndrome, including hemangioblastomas in the brain, spinal cord, or retina, as well as renal cell carcinomas and other neoplasms, can significantly impact quality of life and survival. The prognosis varies depending on the size, location, and response to treatment of these tumors [1].

The treatment of hemangioblastomas often involves the use of laser therapy to target and seal off abnormal blood vessels, thereby preventing further growth and reducing the risk of complications. Laser therapy has been highlighted in various studies as a valuable approach in managing hemangioblastomas. For instance, Utsuki et al. (2011) discussed the utility of intraoperative fluorescent diagnosis of residual hemangioblastoma using 5-aminolevulinic acid, where a laser beam was focused on the tumor during surgery [5]. Similarly, Gläsker et al. (2020) mentioned that laser coagulation or cryotherapy can effectively control peripheral retinal hemangioblastomas [6]. Furthermore, Seifert et al. (1990) emphasized the use of microsurgical removal aided by laser energy as the preferred method for treating spinal hemangioblastomas [7].

Moreover, laser photocoagulation has been identified as a primary treatment option for retinal hemangioblastomas in several studies. Valdés-Lara et al. (2020) recommended laser treatment as the primary approach for capillary hemangioblastomas, especially when combined with antiangiogenics for significant exudation [8]. Additionally, Lang et al. (2017) and Chou et al. (2018) discussed the use of laser photocoagulation in treating hemangioblastomas in the peripheral retina, with Lang et al. (2017) specifically mentioning the postoperative monitoring of hemangioblastomas using optical coherence tomography angiography [9, 10].

The tumors associated with VHL syndrome, such as hemangioblastomas (in the brain, spinal cord, or retina), renal cell carcinomas, and other neoplasms, can significantly impact quality of life and survival depending on their size, location, and response to treatment. Early diagnosis through genetic testing and regular screening is crucial for optimizing outcomes. Prompt intervention, such as surgical resection, laser therapy for retinal hemangioblastomas, and surveillance for cancers, can help manage symptoms and reduce the risk of complications.

## Conclusions

Early identification of VHL syndrome through genetic testing and systematic surveillance is essential for optimizing clinical outcomes. Timely interventions, including surgical resection of neoplasms and laser therapy for retinal hemangioblastomas, are critical in mitigating disease progression. Furthermore, continuous lifelong monitoring for associated malignancies is indispensable to reducing morbidity and improving long-term prognosis in affected individuals.
